# Novel Colloidal Nanocarrier of Cetylpyridinium Chloride: Antifungal Activities on *Candida* Species and Cytotoxic Potential on Murine Fibroblasts

**DOI:** 10.3390/jof6040218

**Published:** 2020-10-12

**Authors:** Heitor Ceolin Araujo, Laís Salomão Arias, Anne Caroline Morais Caldeirão, Lanay Caroline de Freitas Assumpção, Marcela Grigoletto Morceli, Francisco Nunes de Souza Neto, Emerson Rodrigues de Camargo, Sandra Helena Penha Oliveira, Juliano Pelim Pessan, Douglas Roberto Monteiro

**Affiliations:** 1 Department of Preventive and Restorative Dentistry, School of Dentistry, Araçatuba, São Paulo State University (UNESP), Araçatuba SP 16015-050, Brazil; heitorceolin@hotmail.com (H.C.A.); laisarias@hotmail.com (L.S.A.); francisco_nsn@yahoo.com.br (F.N.d.S.N.); juliano.pessan@unesp.br (J.P.P.); 2 Graduate Program in Dentistry (GPD—Master’s Degree), University of Western São Paulo (UNOESTE), Presidente Prudente SP 19050-920, Brazil; annemcaldeirao@gmail.com; 3 School of Dentistry, Presidente Prudente, University of Western São Paulo (UNOESTE), Presidente Prudente SP 19050-920, Brazil; lanay.carol@gmail.com (L.C.d.F.A.); marcela_morceli@hotmail.com (M.G.M.); 4 Department of Chemistry, Federal University of São Carlos (UFSCar), São Carlos SP 13565-905, Brazil; camargo@ufscar.br; 5 Department of Basic Sciences, School of Dentistry, São Paulo State University (UNESP), Araçatuba SP 16015-050, Brazil; sandra.hp.oliveira@unesp.br

**Keywords:** antifungal agents, biofilms, *Candida albicans*, *Candida glabrata*, cetylpyridinium chloride, chitosan, iron oxide nanoparticles

## Abstract

Nanocarriers have been used as alternative tools to overcome the resistance of *Candida* species to conventional treatments. This study prepared a nanocarrier of cetylpyridinium chloride (CPC) using iron oxide nanoparticles (IONPs) conjugated with chitosan (CS), and assessed its antifungal and cytotoxic effects. CPC was immobilized on CS-coated IONPs, and the nanocarrier was physico-chemically characterized. Antifungal effects were determined on planktonic cells of *Candida albicans* and *Candida glabrata* (by minimum inhibitory concentration (MIC) assays) and on single- and dual-species biofilms of these strains (by quantification of cultivable cells, total biomass and metabolic activity). Murine fibroblasts were exposed to different concentrations of the nanocarrier, and the cytotoxic effect was evaluated by MTT reduction assay. Characterization methods confirmed the presence of a nanocarrier smaller than 313 nm. IONPs-CS-CPC and free CPC showed the same MIC values (0.78 µg mL^−1^). CPC-containing nanocarrier at 78 µg mL^−1^ significantly reduced the number of cultivable cells for all biofilms, surpassing the effect promoted by free CPC. For total biomass, metabolic activity, and cytotoxic effects, the nanocarrier and free CPC produced statistically similar outcomes. In conclusion, the IONPs-CS-CPC nanocarrier was more effective than CPC in reducing the cultivable cells of *Candida* biofilms without increasing the cytotoxic effects of CPC, and may be a useful tool for the treatment of oral fungal infections.

## 1. Introduction

*Candida albicans* is a commensal microorganism that may be isolated from oral, vaginal and gastrointestinal microbiomes in most healthy individuals [1]. In cases of unbalanced immune system, however, this fungus may act opportunistically forming biofilms and leading to oral or systemic infections associated with high rates of morbidity and mortality [2]. In oral fungal infections such as denture stomatitis and oropharyngeal candidiasis, *Candida glabrata* is the second or third most prevalent *Candida* species, and exerts interactions of competition [3], antagonism [4] or indifference [5] with *C. albicans*, which may modulate the virulence factors of these species. A cooperative behavior may also be observed, since *C. glabrata* adheres to hyphae of *C. albicans* in order to facilitate its invasion into deeper tissues of the host [6,7].

Cetylpyridinium chloride (CPC) is a quaternary ammonium compound used in mouth-rinses and topical formulations, presenting antifungal activity [8,9,10]. Its effects have been shown to be superior to miconazole [9], also presenting high efficacy in reducing *C. albicans* adhesion to the oral mucosa [11]. However, the low substantivity of CPC [12] can be considered as a drawback of therapies involving this agent due to the need for frequent exposure, which may result in antimicrobial resistance by some *Candida* strains [13,14]. Furthermore, CPC at high concentrations produce significant reductions in the viability of human gingival fibroblasts, regardless of the treatment period (24–72 h) [15]. As for murine fibroblasts and human keratinocytes, even at low concentrations, CPC was cytotoxic after 15 and 60 min of exposure [16].

In response to this scenario, antimicrobial agents have been redesigned to make them more effective against microorganisms and less toxic to human cells. In the field of nanotechnology, nanoparticles of silver [17,18], gold [19], zinc oxide [20] and chitosan [21], applied alone or in combination with other compounds, have shown interesting results in managing *Candida* species. Iron oxide nanoparticles (IONPs) have also been used in several biomedical applications (e.g., drug delivery, tissue engineering, contrast agents) due to their nano-sized and magnetic properties [22,23]. Literature data show that IONPs-based nanocarriers were able to improve the physico-chemical properties of the aggregated drugs and to combat yeasts, bacteria and spores [24,25,26,27,28,29]. Moreover, in order to avoid the aggregation of IONPs and to increase their biocompatibility, coatings with natural polymers such as chitosan (CS) have been adopted in the nanocarrier engineering, forming associations known as core-shell [30,31].

Faced with the aforementioned CPC limitations, the objectives of the present study were to develop and characterize a novel nanocarrier of CPC based on a core-shell association of IONPs and CS, as well as to assess its antifungal and cytotoxic effects using *Candida* species and murine fibroblasts, respectively. The null hypothesis was that the antifungal and cytotoxic effects of the nanocarrier would not differ from those found for the CPC alone.

## 2. Materials and Methods 

### 2.1. Experimental Design

For the current study, the IONPs-CS-CPC nanocarrier was prepared by loading CPC on CS-coated IONPs. Characterization tests using transmission electron microscopy (TEM), dynamic light scattering (DLS), X-ray powder diffraction (XRD), Fourier-transform infrared spectroscopy (FTIR) and thermogravimetric analysis (TGA) were performed in order to determine the shape and size of the particles, the type of crystalline structure, the main functional chemical groups, and the thermal behavior of the nanocarrier. Then the minimum inhibitory concentrations (MICs) of IONPs-CS-CPC against planktonic cells of *C. albicans* and *C. glabrata* were determined. In addition, the antifungal effects of the CPC-containing nanocarrier at 15.6, 39 and 78 µg mL^−1^ on single- and dual-species biofilms of *C. albicans* and *C. glabrata* were evaluated by quantification of cultivable cells, biofilm biomass and metabolic activity. The cytotoxic potential of the nanocarrier on murine fibroblasts was investigated by MTT assay.

### 2.2. Assembly and Characterization of the IONPs-CS-CPC Nanocarrier

The assembly of the IONPs-CS-CPC nanocarrier involved two distinct stages: (i) coating IONPs with CS in a simple mixing process, as previously detailed [24]; (ii) incorporation and solubilization of CPC (1000 µg) (Sigma-Aldrich, St. Louis, MO, USA) in 700 µg mL^−1^ of CS-coated IONPs under magnetic stirring for 1 h. In order to characterize the colloidal nanocarrier, TEM, DLS, XRD, FTIR and TGA were performed following protocols previously described [24].

### 2.3. Candida Strains and Growth Conditions

Two reference strains were evaluated, both from American Type Culture Collection (ATCC): *C. albicans* (ATCC 10231) and *C. glabrata* (ATCC 90030). Stock cultures of each strain at −80 °C were reactivated on Sabouraud Dextrose Agar (SDA; Difco, Le Pont de Claix, France) and, subsequently, suspended in Sabouraud Dextrose Broth (Difco) under aerobic conditions at 37 °C. After incubation for 20–22 h, the inoculum of each strain was adjusted in artificial saliva [32] to 10^7^ cells mL^−1^, according to the protocol described by Monteiro et al. [33].

### 2.4. Antifungal Effect of the IONPs-CS-CPC Nanocarrier

The antifungal effect of the nanocarrier was evaluated on *C. albicans* and *C. glabrata* in their planktonic states or when forming biofilms. For planktonic susceptibility, the standard broth microdilution method in 96-well plates (kasvi, São José dos Pinhais, PR, Brazil) was used to find the minimum inhibitory concentration (MIC). Briefly, CPC-containing nanocarrier at 1000 µg mL^−1^ was diluted in geometric progression in the Roswell Park Memorial Institute (RPMI) 1640 (Sigma-Aldrich) culture medium, resulting in concentrations ranging from 200 to 0.39 µg mL^−1^. Next, 100 µL of each dilution was mixed with an equal volume of the inoculum of each *Candida* species at 2.5–3.0 × 10^3^ colony-forming units (CFUs) mL^−1^ in RPMI 1640. MIC values (100% inhibition of visible fungal growth) were determined after 48 h of incubation at 37 °C. MICs of CPC, CS and IONPs alone were also determined. The analysis of antifungal synergy among the nanocarrier compounds was obtained by calculating the fractional inhibitory concentration index (FICI) in checkerboard assays, according to the criteria reported in detail by Wei and Bobek [34].

For biofilm susceptibility testing, 96-well microtiter plates containing 200 µL of the inoculum (10^7^ cells mL^−1^ in artificial saliva) of each *Candida* species in single culture were incubated for 48 h at 37 °C. For dual-species biofilms, 100 µL of the inoculum of each strain at 2 × 10^7^ cells mL^−1^ (both at final concentration of 1 × 10^7^ cells mL^−1^) were mixed in 96-well plates and then incubated during 48 h to form biofilms. For both single- and dual-species cultures, biofilm refreshment was performed after the first 24 h of incubation. After biofilm formation period (48-h), artificial saliva was removed, and the resulting biofilms were washed with phosphate buffered saline (0.1 M; pH 7.0) to remove non-adherent cells. Biofilm-containing wells were then filled with 200 µL of the colloidal nanocarrier diluted in artificial saliva to attain CPC concentrations of 15.6 (IONPs-CS-CPC15.6), 39 (IONPs-CS-CPC39) and 78 µg mL^−1^ (IONPs-CS-CPC78). These concentrations denote values 20-, 50- and 100-fold times the nanocarrier MIC values. Treatments with CPC (78 µg mL^−1^), CS (54.6 µg mL^−1^) and IONPs (54.6 µg mL^−1^) alone were also performed. As a negative control (NC), biofilms were treated with pure artificial saliva.

After 24 h of treatment at 37 °C with the different compounds, the number of CFUs was counted using SDA and CHROMagar *Candida* (Difco), respectively for single- and dual-species biofilms. Total biomass and metabolic activity quantifications were also performed using the well-established colorimetric methods of crystal violet staining and XTT reduction, respectively, which were detailed in previous studies [35,36]. Quantitative results were represented according to the well area (log_10_ CFU cm^−2^ and absorbance cm^−2^). All microbiological assays (MIC and biofilms) were conducted on three different occasions, each in triplicate.

### 2.5. Cytotoxic Effect of the IONPs-CS-CPC Nanocarrier

Mouse L929 fibroblast cells from ATCC were employed for cytotoxicity testing. These cells were cultured in Dulbecco’s Modified Eagle’s Medium (DMEM; Gibco, Invitrogen Life Technologies, Carlsbad, CA, USA) and incubated under ideal conditions of humidity and temperature, as reported by Takamiya et al. [37]. Briefly, after reaching 90–100% confluence, fibroblast cells were seeded in 24-well plates at 1 × 10^5^ cells well^−1^ and incubated for 24 h. Subsequently, the cell monolayer was exposed to different concentrations (0.24–500 μg mL^−1^) of pure CPC and IONPs-CS-CPC during 24 or 48 h. In order to assess the cytotoxicity after each exposure period, MTT assay was performed, as detailed elsewhere [38]. The results were expressed as % cell viability in relation to the NC group (fibroblasts exposed to pure DMEM).

### 2.6. Statistical Analysis

All biofilm data showed normal distribution (Shapiro-Wilk test) and were statistically examined by 1-way ANOVA followed by Fisher LSD’s test. In turn, cytotoxicity data showed normal and non-normal distribution, respectively for pure CPC (results transformed into square root) and IONPs-CS-CPC and were submitted to 2-way ANOVA followed by Tukey’s test. SigmaPlot software (version 12.0; Systat Software Inc., San Jose, CA, USA) was used in data analysis, adopting a significance level of 5%.

## 3. Results

### 3.1. Characterization of the IONPs-CS-CPC Nanocarrier

**Figure 1:**
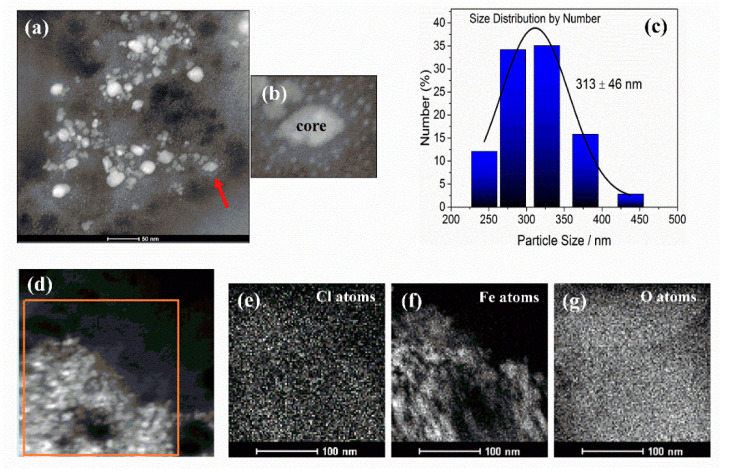
Characterization of the iron oxide nanoparticles-chitosan-cetylpyridinium chloride nanocarrier. Transmission electron microscopy images (**a**,**b**,**d**) and size distribution histogram obtained by dynamic light scattering (**c**) for the nanocarrier. Image “b” represents an increase in the area highlighted with the red arrow in image “a”, where it is possible to clearly observe the cetylpyridinium chloride particles adhered to the core of iron oxide nanoparticles coated with chitosan. The orange area in image (**d**) was analyzed by X-ray energy-dispersive spectroscopy and allowed the identification of chlorine (**e**), iron (**f**) and oxygen (**g**) atoms.

The characterizations of the colloidal suspension of pure IONPs and the IONPs-CS compound have been previously reported [24]. According to Figure 1a, a predominantly spherical shape was found for IONPs and CPC in the nanocarrier, which showed a diameter lower than 50 nm. It was also possible to visualize the IONP core coated with CS, as well as CPC particles attached to CS, forming a core-shell nanocarrier (Figure 1a,b). By using DLS, however, the average hydrodynamic diameter of the nanocarrier was 313 ± 46 nm (Figure 1c). Chlorine (Cl; Figure 1e), iron (Fe; Figure 1f) and oxygen (O; Figure 1g) atoms were identified by energy-dispersive spectroscopy (EDS) in the nanocarrier image represented in Figure 1d, confirming the presence of CPC, IONPs and CS in the sample.

The XRD results found for IONPs-CS-CPC were similar to those seen for a chlorhexidine nanocarrier developed by our group [24]. In general, the positions and widths observed for diffractograms of IONPs, IONPs-CS and IONPs-CS-CPC were equivalent, demonstrating that the assembly of the nanocarrier did not structurally affect the IONPs. In addition, diffractograms revealed the presence of nanoparticles with spinel-like crystalline structure [24].

**Figure 2:**
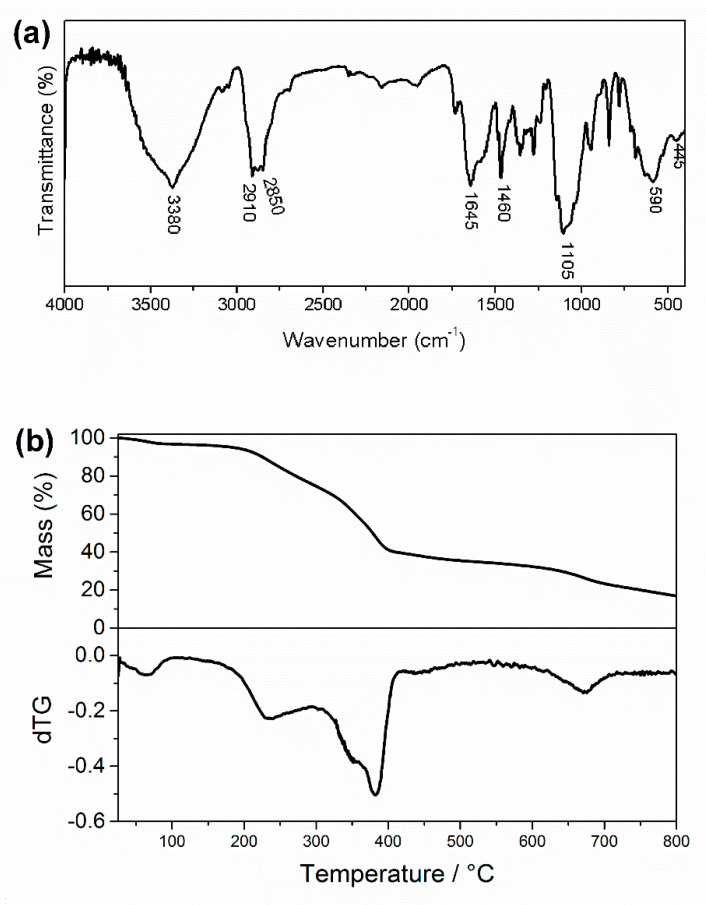
Characterization of the iron oxide nanoparticles-chitosan-cetylpyridinium chloride nanocarrier. Fourier-transform infrared spectra (**a**) and thermogravimetric curve (**b**) obtained for the nanocarrier.

Absorption peaks representing the main functional groups of IONPs and CS [24] were also detected in the FTIR spectrum of the IONPs-CS-CPC nanocarrier (Figure 2a). Furthermore, asymmetric and symmetrical stretching vibrations of C–H (ʋC_sp_^3^–H) were represented by bands around 2910 and 2850 cm^−1^, respectively, which are typical of the CPC methylene chain (Figure 2a) [39]. According to TGA, the total decomposition of the CPC present in the nanocarrier occurred at temperatures up to 400 °C (Figure 2b). Finally, as the final mass loss of the nanocarrier at 800 °C was similar to that found for IONPs and IONPs-CS [24], it can be assumed that the binding of CPC to IONPs-CS was successful.

### 3.2. Antifungal Effect of the IONPs-CS-CPC Nanocarrier

For the two strains evaluated, CPC, either free or forming the nanocarrier, showed the same MIC values (0.78 µg mL^−1^). Thus, the effect of the interaction among components of the nanocarrier on planktonic cells was classified as indifferent (Table 1).

**Figure 3:**
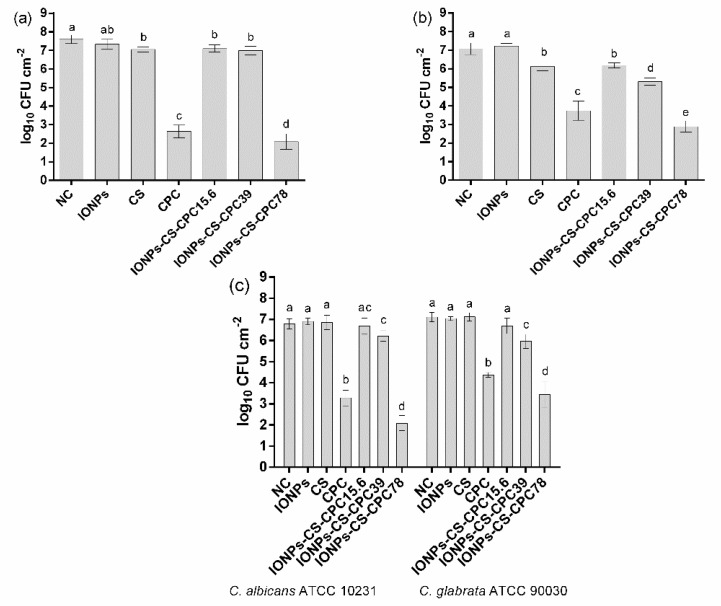
Quantification of biofilm cultivable cells. Mean values of the logarithm of colony-forming units per cm^2^ for single biofilms of *Candida albicans* ATCC 10231 (**a**) *Candida glabrata* ATCC 90090 (**b**) and dual-species biofilms (**c**) treated with 54.6 μg mL^−1^ iron oxide nanoparticles (IONPs), 54.6 μg mL^−1^ chitosan (CS), 78 μg mL^−1^ cetylpiridinium chloride (CPC) and nanocarrier containing CPC at 15.6 (IONPs-CS-CPC15.6), 39 (IONPs-CS-CPC39) and 78 μg mL^−1^ (IONPs-CS-CPC78). Negative control (NC) indicates biofilms treated with pure artificial saliva (without drugs). Error bars represent standard deviations of the means. Different lower case letters indicate significant differences among the groups by one-way ANOVA and Fisher LSD’s test (*p* < 0.05).

Concerning the effect on single-species biofilms, all tested compounds significantly reduced the number of CFUs compared to NCs, except for treatment with IONPs alone (Figure 3a,b). IONPs-CS-CPC78 was the most effective treatment, statistically differing from the other groups and achieving reductions of 5.51- and 4.18-log_10_ compared to controls, respectively for *C. albicans* and *C. glabrata* (Figure 3a,b). As for dual-species biofilms, IONPs-CS-CPC78 also differed from the other groups and promoted the highest decreases in CFUs compared to NC, with values of 4.71- and 3.67-log_10_, respectively for *C. albicans* and *C. glabrata* (Figure 3c).

**Figure 4:**
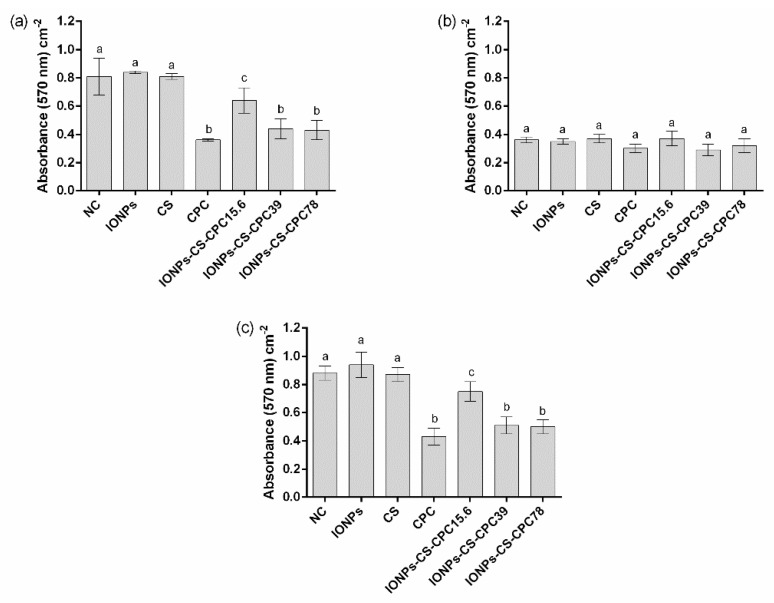
Quantification of total biofilm biomass by crystal violet staining. Mean absorbance values per cm^2^ for single biofilms of *Candida albicans* ATCC 10231 (**a**), *Candida glabrata* ATCC 90030 (**b**), and dual-species biofilms (**c**) treated with 54.6 μg mL^−1^ iron oxide nanoparticles (IONPs), 54.6 μg mL^−1^ chitosan (CS), 78 μg mL^−1^ cetylpyridinium chloride (CPC) and nanocarrier containing CPC at 15.6 (IONPs-CS-CPC15.6), 39 (IONPs-CS-CPC39) and 78 μg mL^−1^ (IONPs-CS-CPC78). Negative control (NC) indicates biofilms treated with pure artificial saliva (without drugs). Error bars represent standard deviations of the means. Different lower case letters indicate significant differences among the groups by one-way ANOVA and Fisher LSD’s test (*p* < 0.05).

Total biomass results showed that CPC, IONPs-CS-CPC39 and IONPs-CS-CPC78 did not differ from each other, and resulted in the highest reductions compared to NCs, both for single biofilm of *C. albicans* (Figure 4a) and dual-species biofilm (Figure 4c). These significant reductions ranged from 42 to 55%. For single biofilm of *C. glabrata*, however, no significant reduction in biomass was found after exposure to the different compounds (Figure 4b).

**Figure 5:**
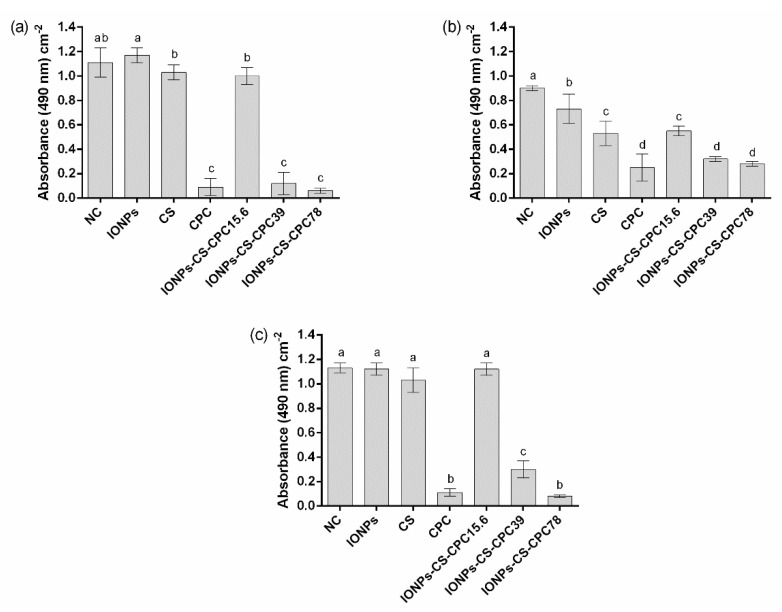
Quantification of biofilm metabolic activity by XTT reduction assay. Mean absorbance values per cm^2^ for single biofilms of *Candida albicans* ATCC 10231 (**a**), *Candida glabrata* ATCC 90030 (**b**), and dual-species biofilms (**c**) treated with 54.6 μg mL^−1^ iron oxide nanoparticles (IONPs), 54.6 μg mL^−1^ chitosan (CS), 78 μg mL^−1^ cetylpyridinium chloride (CPC) and nanocarrier containing CPC at 15.6 (IONPs-CS-CPC15.6), 39 (IONPs-CS-CPC39) and 78 μg mL^−1^ (IONPs-CS-CPC78). Negative control (NC) indicates biofilms treated with pure artificial saliva (without drugs). Error bars represent standard deviations of the means. Different lower case letters indicate significant differences among the groups by one-way ANOVA and Fisher LSD’s test (*p* < 0.05).

For both single biofilms of *C. albicans* (Figure 5a) and *C. glabrata* (Figure 5b), CPC, IONPs-CS-CPC39 and IONPs-CS-CPC78 significantly reduced the metabolic activity compared to NCs. For the dual-species biofilm, a similar trend was observed, except that CPC and IONPs-CS-CPC78 led to the highest reductions (Figure 5c).

### 3.3. Cytotoxic Effect of the IONPs-CS-CPC Nanocarrier

**Figure 6:**
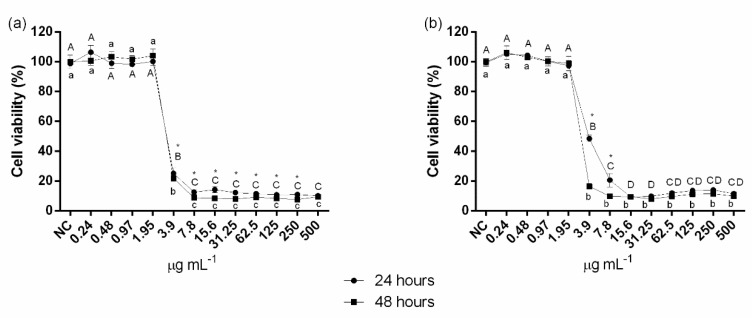
Cytotoxic potential evaluated by MTT reduction assay. Cell viability percentages of L929 murine fibroblasts after exposure for 24 or 48 h to different concentrations (0.24–500 μg mL^−1^) of cetylpyridinium chloride (**a**) and iron oxide nanoparticles-chitosan-cetylpyridinium chloride nanocarrier (**b**). Negative control (NC) represents murine fibroblasts exposed to pure culture medium (without drugs). Error bars represent standard deviations of the means. Different uppercase and lowercase letters indicate significant differences among the different concentrations of the compounds, respectively for 24 and 48 h of exposure. *Indicates significant difference between 24 and 48 h of exposure, within each concentration tested (two-way ANOVA followed by Tukey’s test; *p* < 0.05).

CPC was not cytotoxic at concentrations equal to or lower than 1.95 μg mL^−1^, either in the free state or forming the nanocarrier, regardless of the exposure period evaluated (Figure 6a,b). However, for free CPC, concentrations equal to or higher than 3.9 μg mL^−1^ promoted significant reductions in L929 cell viability, ranging from 74.8–89.9% and 78.1–90.5%, respectively for 24 and 48 h of exposure (Figure 6a). In turn, the CPC-containing nanocarrier at the same concentration (3.9 μg mL^−1^) led to significant reductions in cell viability of 51.5% and 83.7%, respectively for 24 and 48 h of exposure (Figure 6b). The nanocarrier at CPC concentrations > 7.8 μg mL^−1^ further induced to higher levels of toxicity (fibroblast viability < 11.4%; Figure 6b).

Comparisons between 24 and 48 h of exposure for each concentration revealed that free CPC at 3.9–250 μg mL^−1^ was significantly more cytotoxic after 48 h of exposure (Figure 6a). For the nanocarrier, CPC only at 3.9 and 7.8 μg mL^−1^ induced to more cytotoxicity after 48 h of exposure (Figure 6b).

## 4. Discussion

In the present study, the IONPs-CS-CPC nanocarrier was significantly more effective in reducing the number of CFUs in biofilms, despite promoting similar effects to those of CPC on planktonic cells and murine fibroblasts. Thus, the study’s null hypothesis was partially rejected.

**Figure 7:**
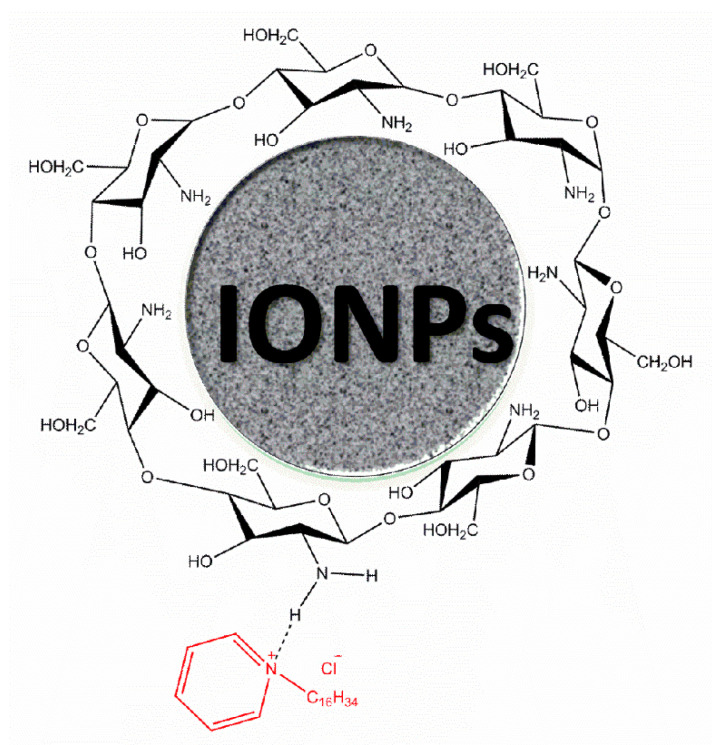
Schematic representation of the possible chemical bonds/interactions among the compounds that form the iron oxide nanoparticles-chitosan-cetylpyridinium chloride nanocarrier. A bond between oxygen from iron oxide nanoparticles (IONPs) and hydrogen from amine group (-NH_2_) of chitosan (CS) is responsible for coating IONPs with CS. In turn, cetylpyridinium chloride (shown in red) establishes a hydrogen interaction with the amine group of CS.

Regarding the assembly of the nanocarrier, the characterization results showed that CS successfully coated the IONPs, while all the added CPC remained anchored on the core-shell structure (IONPs-CS) (Figure 1 and Figure 2). IONPs display advantages that allow their use in drug delivery systems, such as the largest ratio of surface area to volume due to their nanometric size. In addition, these nanoparticles have grafted ligands and portions of selective binding sites that favor the binding of both natural biomolecules and other compounds (organic or inorganic) used as shell [40]. In turn, CS has been widely employed as shell for IONPs, as it prevents the agglomeration of nanoparticles (i.e., acting as a stabilizing agent), and limits nonspecific cell uptake, in addition to enabling the immobilization of different drugs [41]. For the IONPs-CS-CPC nanocarrier, a strong bond between hydrogen of the primary amino group (-NH_2_) of CS and oxygen of IONPs is the likely mechanism responsible for the conjugation of IONPs with CS [42]. It is also believed that CPC forms a hydrogen interaction with the amino group of CS, which is protonated in an acidic medium (Figure 7). Interestingly, despite the fact that a carrier system of nanometric dimensions was formed, larger sizes were observed by DLS compared to TEM. Such differences may be explained by the methodological principles that make up each technique. For TEM, the electron beam crosses through an ultrafine and dry sample [43], while in the indirect DLS technique the particle size is obtained through the frequency of movement in an aqueous medium, which may generate clusters of particles [44].

Although previous studies have shown that carriers based on IONPs improve the effect of some drugs on bacterial and fungal planktonic cells [28,45,46], the present study found equal MIC values for CPC alone or conjugated to the IONPs-CS complex (Table 1). This demonstrates that the additional antimicrobial effects promoted by the nanocarriers may be associated with the type of immobilized drug and microorganism tested. In addition, FICI values classified the interaction among the compounds present in the IONPs-CS-CPC nanocarrier as indifferent (Table 1), suggesting that its effect on *Candida* planktonic cells is primarily dependent on the presence of CPC itself. CPC is formed by a hydrophilic region, which interacts with microbial cell surfaces, including the cytoplasmic membrane [47]. As recently reported, the interaction between the negative charge of the microorganism with the pyridinium ion (positively charged) may trigger changes in the lipid membrane [48]. Furthermore, at low concentrations, CPC impairs cell osmoregulation and homeostasis, while at high concentrations it promotes leakage of cytoplasmic content due to the disintegration of the cell membrane [48].

For biofilm analyzes, the IONPs-CS-CPC78 nanocarrier was more effective in reducing the number of CFUs of single- and dual-species biofilms than free CPC (Figure 3). For CPC alone, an effect limited to the middle and outer layers of biofilms has been reported [49], probably due to electrostatic or hydrophobic interactions between extracellular matrix and CPC, which hinders drug penetration [48]. Consequently, it seems rational that the favorable results found in the present study are related to the greater ease of the nanostructure in penetrating into the deeper layers of *Candida* biofilms, which allowed the conjugated CPC to act in broader and deeper areas of biofilms compared to free CPC. In fact, previous studies by EDS mapping have shown the presence of Fe atoms (characteristic of IONPs) well distributed on *Candida* biofilms and on polystyrene surfaces, proving the effectiveness of IONPs as drug carriers [24,28]. The results also revealed that IONPs alone did not lead to significant reductions in CFUs, which could be expected based on previous findings showing that IONPs can either stimulate or inhibit microbial growth depending on the strain and concentration tested [50]. CS, on the other hand, was able to decrease this parameter for single biofilms, which could be associated with its ability to impair cell membrane permeability, resulting in release of cytoplasmic content and death of the microorganisms [51]. Therefore, the effects of the nanocarrier on CFUs may be attributed to a combined action of CS and CPC, with greater emphasis on the latter.

In general, total biomass and metabolic activity results should also be considered as positive, since the nanocarriers at 39 and 78 µg mL^−1^ showed similar effects to those found for pure CPC at 78 µg mL^−1^ (Figure 4 and Figure 5). Another aspect of clinical relevance refers to the mucoadhesive property of CS [52], which might solve the issue of low substantivity observed for CPC [12]. The greater substantivity of CPC in the nanocarrier would reduce the number of applications of the product per day (which may enhance patient’s adherence to the treatment), as well as the emergence and spread of microbial resistance.

As for the analysis in murine fibroblasts, both free CPC and the nanocarrier significantly reduced cell viability at concentrations equal to or higher than 3.9 µg mL^−1^, regardless of the contact period (24 or 48 h) (Figure 6). Considering that the presence of IONPs and CS did not increase the cytotoxic potential of the nanocarrier compared to free CPC, it is possible to assume that the effect on murine fibroblasts was essentially related to CPC. The simultaneous analysis of cytotoxicity results and MIC values evidences that both free CPC or CPC conjugated to the nanocarrier were not cytotoxic at the effective concentration against *Candida* planktonic cells (0.78 µg mL^−1^). However, at the concentrations tested on biofilms (15.6, 39 and 78 µg mL^−1^) both compounds led to significant reductions in L929 cell viability (~90%). Müller et al. [53] also observed that mouthwashes containing CPC had antimicrobial properties but were highly toxic to gingival fibroblasts. In addition, this compound is able to induce apoptosis in pulmonary epithelial cells [54], and to inhibit mitochondrial oxygen consumption and ATP synthesis for different cell cultures [55]. On the other hand, CPC may prevent osteoclastic formation in mouse bone marrow cells, positively contributing to the prevention of bone loss without leading to cytotoxic effects, depending on the concentration assessed [56]. Based on the above, the issues related to CPC’s cytotoxicity (either alone or forming nanocarriers) point to the need for the study of alternatives to reduce possible side effects.

Finally, it is worth emphasizing that the oral cavity is formed by different cell types in addition to fibroblasts, and that *Candida* species coexist with several microorganisms. Consequently, future studies evaluating the performance of the IONPs-CS-CPC nanocarrier on microcosms of oral fungal infections, as well as on reconstituted oral tissues with multiple layers, may provide additional data on the carrier’s actual benefits. Such analyzes, along with the evaluation of the molecular mechanisms of action of the nanocarrier, may help in the development of effective and safe strategies to manage oral candidiasis.

## 5. Conclusions

Summarizing, a nanocarrier of CPC was effectively formed by immobilizing this drug on CS-coated IONPs. The IONPs-CS-CPC nanocarrier was shown to be more effective than pure CPC in reducing the cultivable cells of *Candida* biofilms without increasing the cytotoxic effects of CPC. These results suggest that this new nanocarrier may be a useful tool for the treatment of oral fungal infections. 

## Figures and Tables

**Table 1:** Minimum inhibitory concentration (MIC, µg mL^−1^) values of free cetylpyridinium chloride (CPC) or forming the nanocarrier in conjunction with iron oxide nanoparticles (IONPs) and chitosan (CS), against *Candida* strains.

Species	Free CPC	Forming the Nanocarrier			FICI	Classification
		IONPs	CS	CPC		
*C. albicans* ATCC 10231	0.78	0.54	0.54	0.78	>1.00	Indifference
*C. glabrata* ATCC 90030	0.78	0.54	0.54	0.78	>1.00	Indifference

Note: Free iron oxide nanoparticles (IONPs) and CS resulted in minimum inhibitory concentration (MIC) values higher than 140 µg mL^−1^, as previously reported [28]. FICI - fractional inhibitory concentration indices for the nanocarrier.
